# Novel Treatment Strategies for Hormone Receptor (HR)-Positive, HER2-Negative Metastatic Breast Cancer

**DOI:** 10.3390/jcm13123611

**Published:** 2024-06-20

**Authors:** Antonella Ferro, Michela Campora, Alessia Caldara, Delia De Lisi, Martina Lorenzi, Sara Monteverdi, Raluca Mihai, Alessandra Bisio, Mariachiara Dipasquale, Orazio Caffo, Yari Ciribilli

**Affiliations:** 1 Medical Oncology and Breast Unit, Santa Chiara Hospital, APSS Trento, 38122 Trento, Italy; alessia.caldara@apss.tn.it (A.C.); delia.delisi@apss.tn.it (D.D.L.); martina.lorenzi@apss.tn.it (M.L.); sara.monteverdi@apss.tn.it (S.M.); mariachiara.dipasquale@apss.tn.it (M.D.); 2 Department of Pathology, Santa Chiara Hospital, APSS Trento, 38122 Trento, Italy; michela.campora@apss.tn.it; 3 Department of Pathology, Queen Elizabeth University Hospital, Glasgow G51 4TF, UK; simiuc.raluca@gmail.com; 4 Department of Cellular, Computational and Integrative Biology (CIBIO), University of Trento, 38123 Trento, Italy; alessandra.bisio@unitn.it (A.B.); yari.ciribilli@unitn.it (Y.C.); 5 Medical Oncology, Santa Chiara Hospital, APSS Trento, 38122 Trento, Italy; orazio.caffo@apss.tn.it

**Keywords:** hormone-positive HER2-negative breast cancer, resistance mechanisms to ET and/or CDK4/6i, endocrine therapy, CDK4/6 inhibitors, next-generation endocrine agents, targeted therapies

## Abstract

Estrogen receptor (ER)-positive breast cancer (BC) is the most common BC subtype. Endocrine therapy (ET) targeting ER signaling still remains the mainstay treatment option for hormone receptor (HR)-positive BC either in the early or in advanced setting, including different strategies, such as the suppression of estrogen production or directly blocking the ER pathway through SERMs—selective estrogen receptor modulators—or SERDs—selective estrogen receptor degraders. Nevertheless, the development of de novo or acquired endocrine resistance still remains challenging for oncologists. The use of novel ET combined with targeted drugs, such as cyclin-dependent kinase 4 and 6 (CDK4/6) inhibitors, has significantly improved long-term outcome rates, thus changing the therapeutic algorithm for metastatic BC (MBC) and recently the therapeutic strategy in the adjuvant setting for early high-risk BC. Eluding the resistance to CDK4/6 inhibitors combined with ET is currently an unmet medical need, and there is disagreement concerning the best course of action for patients who continue to progress after this combination approach. Genetic changes in the tumor along its growth uncovered by genomic profiling of recurrent and/or metastatic lesions through tumor and/or liquid biopsies may predict the response or resistance to specific agents, suggesting the best therapeutic strategy for each patient by targeting the altered ER-dependent pathway (novel oral SERDs and a new generation of anti-estrogen agents) or alternative ER-independent signaling pathways such as PI3K/AKT/mTOR or tyrosine kinase receptors (*HER2* mutations or HER2 low status) or by inhibiting pathways weakened through germline *BRCA1/2* mutations. These agents are being investigated as single molecules and in combination with other target therapies, offering promising weapons to overcome or avoid treatment failure and propose increasingly more personalized treatment approaches. This review presents novel insights into ET and other targeted therapies for managing metastatic HR^+^/HER2^−^ BC by exploring potential strategies based on clinical evidence and genomic profiling following the failure of the CDK4/6i and ET combination.

## 1. Introduction

Breast cancer (BC) is the most common cancer diagnosed globally, accounting for 24.5% of all newly diagnosed malignancies [1]. BC mortality has been significantly reduced by early detection brought forward by mammographic screening and by improved adjuvant therapy options for localized disease [1]. An approximate of 70–80% of all cases of BC that are detected are positive for the estrogen receptor (ER) [2], and 65% of those cases are also positive for the progesterone receptor (PR) [3].

These tumors can be made more sensitive to hormonal manipulation with a variety drugs, including by directly blocking the ER pathway with selective estrogen receptor modulators (SERMs), such as tamoxifen, or with selective estrogen receptor degraders (SERDs), such as fulvestrant, or by suppressing the production of estrogen with aromatase inhibitors (AIs).

The progression-free survival (PFS) and, in some cases, overall survival (OS) of patients diagnosed with invasive BC has dramatically improved with the introduction of inhibitors of cyclin-dependent kinases 4 and 6 (CDK4/6is). CDK4/6is act as cell cycle blockers by suppressing the downstream effects of the complex formed by CDK4/6 with cyclin D, and induce cell cycle arrest in G1 phase, thereby preventing entry into S phase and subsequent DNA synthesis [4].

While CDK4/6i addition to endocrine therapy (ET) has been established as the front-line standard-of-care for HR+/HER2-negative MBC, the optimal sequence post-CDK4/6i strategy remains controversial.

### CDK4/6is: Indications and Use

In the PALOMA-2, MONALEESA-2, MONARCH-3, and MONALEESA-7 phase III trials, all three CDK4/6is (palbociclib, ribociclib, and abemaciclib) in combination with ET demonstrated homogeneous improvements in PFS as first-line treatments in both post-menopausal and pre-menopausal women [5,6,7,8].

Although all first-line studies of different CDK4/6is combined with ET showed similar relative benefits in PFS, a significant improvement in OS has so far been reported for ribociclib combined with ET in pre- and post-menopausal women in both the MONALEESA-7 and -2 trials [9,10]. On the other hand, in the MONARCH-3 trial, abemaciclib reached a numerical (the observed improvement in median OS was 13.1 months), but not statistically significant, improvement in OS at its final analysis [11].

Conversely, no significant OS benefit was observed with palbociclib as a first-line treatment in the PALOMA-2 trial; it remains unclear if this is due to the following factors:

(i) The differential efficacy of various CDK4/6is. Effectively, palbociclib, ribociclib, and abemaciclib showed similar pharmacokinetics, but variable pharmacodynamics (ribociclib and abemaciclib are more selective toward CDK4 over CDK6; abemaciclib has a different chemical structure and additional inhibitory activity toward multiple kinases). These characteristics could explain the differences in toxicity, half-life, ability to cross the blood–brain barrier, acquired resistance mechanisms, and, ultimately, efficacy;
(ii) Differences in study populations;
(iii) Missing survival data;
(iv) Treatment discontinuation for limiting adverse events (AEs) [12].

The benefit of CDK4/6is has been confirmed in all studies, regardless of the endocrine backbone (aromatase inhibitors or fulvestrant), age, number and/or types of metastatic sites, cancer subtypes (lobular or ductal), the length of the treatment-free interval, or the line of therapy.

Therefore, current ASCO (American Society of Clinical Oncology) and ESMO (European Society for Medical Oncology) guidelines agree in recommending CDK 4/6is combined with ET as the standard-of-care in the first-line treatment of HR^+^/HER2-negative MBC, unless the patient has imminent organ failure or a life-threatening visceral crisis; in this case, chemotherapy (CT) is considered the best approach to rapidly control the disease. The RIGHT Choice is the first prospective phase II trial evaluating the first head-to-head comparison of a CDK4/6i (ribociclib) and ET combination with combination CT in pre-menopausal patients with HR^+^, HER2^−^ aggressive MBC, including symptomatic visceral metastases, rapid disease progression, impending visceral compromise, or markedly symptomatic non-visceral disease. The outcomes of the trial showed that ribociclib and ET had about a one year longer PFS (24.0 vs. 12.3 months; HR 0.54; CI: 0.36–0.79; *p* = 0.0007) and time to failure (18.6 vs. 8.5 months; HR 0.45; 95% CI: 0.56–1.09) compared to combination CT. Noteworthy, the time to onset of the response and overall response rate (ORR) were similar between the two treatment groups [13]. Furthermore, ribociclib combined with ET appeared to be as effective in younger patients (under 40 years) as in those ≥40 years [14]. These findings suggest the potential role of CDK4/6is in the management of aggressive and symptomatic HR^+^/HER2^−^ MBC, offering more effective treatment options in a visceral crisis scenario.

The combination of CDK 4/6is with fulvestrant has also shown efficacy after relapse or progression on an AI as second-line ET (Table 1).

The CDK 4/6i abemaciclib was recently approved in the adjuvant setting based on the results of the MonarchE trial, which reported significant benefits in invasive disease-free survival (iDFS), when it was added to adjuvant AI therapy in patients with HR^+^, HER2^−^ early high-risk BC (5-year iDFS rates: 83.6% vs. 76.0%, HR 0.680; 95% confidence interval 0.599–0.772) [19]. Recently, the NATALEE (NCT03701334) phase III trial has shown an improvement in iDFS with the addition of adjuvant ribociclib to ET compared with ET + placebo in stages II and III (3-year iDFS 90.4% vs. 87.1%, HR for iDFS 0.75; 95% confidence interval, 0.62 to 0.91; *p* = 0.003).

Conversely, palbociclib did not improve outcomes (iDFS) in two other similar clinical trials (PENELOPE-B and PALLAS) [20,21]. These different results could be explained by either different adherence rates (valid for PALLAS, but not for PENELOPE-B), a shorter duration of administration (in PENELOPE-B, palbociclib was administered for 1 year vs. abemaciclib for 2 years in MonarchE), or the lower efficacy of palbociclib compared with the other two CDK4/6is. Despite the initial clinical success of CDK4/6is, the development of resistance to endocrine therapy or to CDK4/6is remains a challenge in the management of HR^+^/HER2^−^ BC patients.

## 2. Mechanisms of Resistance to Endocrine Therapy and CDK4/6 Inhibitors

### 2.1. Resistance to Endocrine Therapy

Different mechanisms of resistance to ET have been described and summarized in several reviews [22,23]. The three main reported processes involve (i) alterations in the estrogen receptor (amplifications, fusions, or mutations in the *ESR1* gene which encodes ERα, often cited in this review as ER), (ii) aberrations in regulators of the ER pathway (i.e., affecting co-factors, chromatin modifiers, or miRNAs), and (iii) changes in other signaling cascades (i.e., hyper-activation of growth factor—GF—receptors).

#### 2.1.1. Alterations in the Estrogen Receptor

Events involving the *ESR1* gene are among the best described alterations associated with resistance to ETs. *ESR1* mutations in the ligand-binding domain are among the most frequent genetic events affecting ERα and resulting in ET failure [24]. Moreover, larger chromosomal aberrations involving the *ESR1* gene were described in ET-resistant BC. These alterations include gene fusions within the same chromosome (*ESR1-CDC107*) or with oncogenes positioned in other chromosomes (*ESR1-YAP1* and *ESR1-PCDH11X*) [25,26]. Additionally, an increase in *ESR1* copy numbers resulting in elevated expression levels of ERα was found in ET-refractory BC with variable rates (1–37%) [27,28,29]. However, the association of *ESR1* amplifications with ET resistance is still debated [30,31], and there are reports linking it to reduced sensitivity to tamoxifen [32], while others report no correlation [33].

#### 2.1.2. Aberrations in ER Pathway Regulators

In the second group of processes negatively influencing the sensitivity to ETs, we emphasize the role of NF1 (encoding for neurofibromin) [34]. Neurofibromin, a GTPase activating protein (GAP), which on one side favors the inactivation of RAS-MAPK signaling pathway and, on the other side, works as a co-repressor for ERα in the promoter region of *CCND1* gene. In this way, the loss of or inactivating mutations in *NF1* lead to amplification of the RAS-dependent signal transduction and, in parallel, to de-repression of cyclin D1, resulting in ET resistance and enhanced proliferation [35,36]. Moreover, *CYP19A1*, the gene encoding the aromatase enzyme that generates estrogen starting from testosterone, was often found over-expressed in ET-resistant BCs that relapsed after AI treatment [37]. Furthermore, alterations (deletions or truncating mutations that are somewhat frequent in BC patients) in the histone H3K4 methyl-transferase KMT2C (also known as MLL3) in ER^+^ MBC patients who received AIs were associated with poorer PFS when compared with *KMT2C* WT ER^+^ MBC patients [38].

#### 2.1.3. Changes in GF Signaling Cascades

Lastly, an aberrant GF signaling pathway can also lead to a reduced sensitivity to ETs in ER^+^ BC. Mainly, the over-activation of these pathways converges to the MAPK- or PI3K/AKT/mTOR-dependent stimulation of cell growth and the ligand-independent activation of ER. Different genetic events were described in BC patients experiencing these effects and usually involve gene mutations and amplifications. On top of the *NF1* alterations that we have mentioned above, amplifications in receptor tyrosine kinases (RTKs) such as EGFR (epidermal growth factor receptor) and, particularly, FGFR1 (fibroblast growth factor receptor 1) were frequent in ET-resistant BC [22]. Interestingly, mutations or amplifications in *ERBB2* gene (encoding HER2) were found in around 5% and 2%, respectively, of ER^+^/HER2^−^ MBC [24]. Mutations in *PIK3CA* gene were among the most frequent event associated with reduced sensitivity to ET (>35%) [39], but mutations in *AKT1* or in *PTEN* were also often described [40]. Additionally, mutations in other components of the RTK-MAPK pathway, like *BRAF*, *RAS*, and *MAP2K1*, were detected in AI-resistant MBC [41].

### 2.2. Resistance to CDK4/6 Inhibitors

Although the use of CDK4/6 inhibitors improved the outcomes of BCs resistant to ET, many different mechanisms of resistance to CDK4/6is have been reported [42,43]. These include (a) alterations in genes controlling cell cycle regulation, (b) the activation of alternative pathways, and (c) changes in transcriptional and epigenetic modifiers.

#### 2.2.1. Alterations in Genes Controlling Cell Cycle Regulation

Specifically, in the first group, the over-expression or amplification of the *CCND1* gene (encoding cyclin D1, the main partner of CDK4/6) was frequently found in BC patients resistant to CDK4/6is [44]. Additionally, the over-expression of the *CCNE1* gene, encoding another cyclin, namely cyclin E1 (the crucial cofactor for CDK2 necessary for RB hyper-phosphorylation), was linked to a limited response to palbociclib in the PALOMA-3 study [45]. Activating mutations in critical domains of CDK4/6 themselves (i.e., the ATP-binding pocket) have also been described to favor resistance [43]. Given that the retinoblastoma protein (RB) is the main phosphorylation target of CDK4/6 to stimulate cell cycle progression, inactivating mutations or the loss of the *RB1* gene was found in different BC patients with reduced sensitivity to CDK4/6 inhibitors [46,47].

#### 2.2.2. Activation of Alternative Pathways

While no association was found between an alteration in the *PIKCA* gene (encoding the catalytic subunit of PI3K, p110) and resistance to CDK4/6is, mutations in *AKT1*, *AURKA*, and *KRAS* genes were found in CDK4/6i-resistant HR^+^/HER2-negative BC. Indeed, activating mutations or amplifications in the *AKT1* and *AKT3* genes correlate with reduced sensitivity to CDK4/6 inhibitors [46]. Moreover, alterations in all three members of the RAS family, such as the recurrent oncogenic mutations KRAS^G12D^, HRAS^K117R^ or amplifications in *NRAS*, were observed in HR^+^/HER2^−^ BC that did not respond well to CDK4/6is [46]. Furthermore, the over-activation of the fibroblast growth factor receptor (FGFR) downstream pathway appeared to be enriched in CDK4/6i-resistant BC. Over-expression or amplifications in FGFR1/2 were also associated with reduced sensitivity to CDK4/6is in BC [48]. Additionally, the loss or inactivating mutations in the *FAT1* tumor suppressor gene, an inhibitor of the Hippo pathway, lead to the enrichment of YAP/TAZ transcription factors on the *CDK6* promoter, resulting in its increased expression and CDK4/6i failure in ER^+^ BC [49].

#### 2.2.3. Changes in Transcriptional and Epigenetic Modifiers

Eventually, in the last group of CDK4/6i resistance mechanisms, we include chromatin remodelers, histone modifiers, and miRNA or lncRNA regulatory circuits. Increased activity of histone deacetylases (HDACs) was associated with CDK4/6i tolerance potentially through the activation of p21-mediated cell cycle arrest/survival [50,51]. Furthermore, a relatively recent report showed that miR-432 was able to elevate CDK6 levels by reducing TGF-β signaling via SMAD4 downregulation, thereby negatively influencing CDK4/6i effectiveness [52]. Moreover, Jin and colleagues proved that the lncRNA TROJAN resulted in the upregulation of CDK2 by binding NKRF (and impeding its inhibitory effect on RELA/p65), hence favoring a reduced sensitivity of ER^+^ BC cells to CDK4/6i [53]. An increased activity of pro-aggressiveness transcription factors such as NF-κB, AP-1, or E2F was also linked to ineffective CDK4/6i treatment in BC [43].

**Figure 1:**
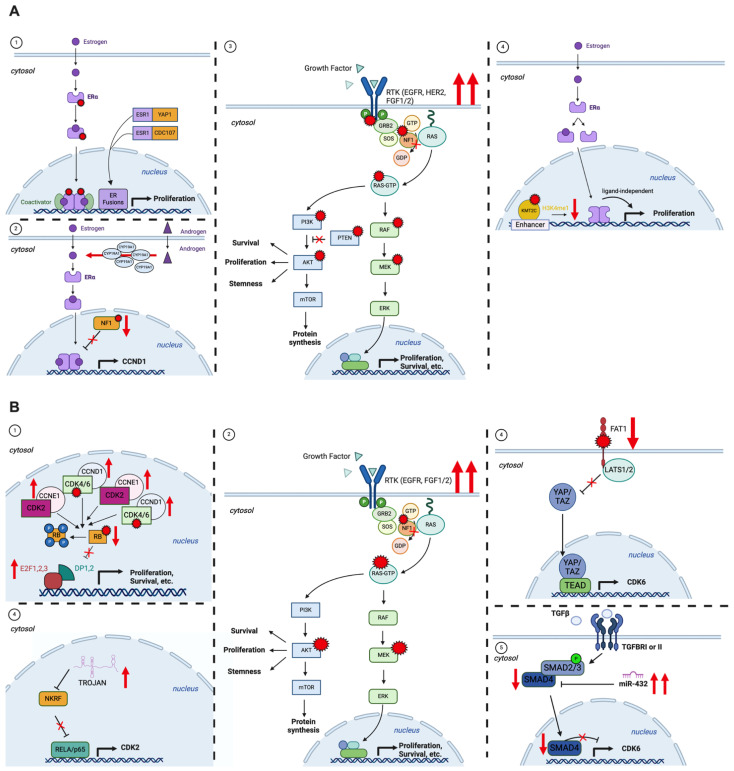
A schematic representation of the main mechanisms of resistance to ET (**A**) and CDK4/6i (**B**) treatments. In red are the most frequent alterations found in HR^+^/HER2^−^ MBC refractory to those therapies. Created with BioRender.com.

We have summarized the main mechanisms of resistance to CDK4/6i and ET treatments in Figure 1.

## 3. Therapeutic Strategies after Progression on CDK4/6i Therapy

After progression on CDK4/6i therapy, no established sequence for the following lines of systemic therapy exists so far. Reasonable options include switching to another ET as a single agent, continuing CDK4/6 inhibitors beyond progression, ET associated with everolimus (a mammalian target of rapamycin, mTOR, inhibitor), a combination of alpelisib (phosphoinositide 3-kinase, PI3K, inhibitor) or capivasertib (AKT pathway inhibitor) with ET for patients with somatic *PIK3CA* mutations, olaparib or talazoparib (poly (ADP-ribose) polymerase, PARP, inhibitors) for patients with germline *BRCA1,2* mutations, or cytotoxic chemotherapy.

Considering the five major randomized trials (MONALEESA-2/7, MONARCH-3, and PALOMA-1/2), patients with progression after first-line treatment with CDK4/6is received single-agent ET in 65% of cases (min–max 48–83%), chemotherapy in 44% (min–max 32–73%) of cases, CDK4/6 inhibitors in up to 38% of cases (on average in 18% of cases), and mTOR inhibitors in 17% of cases (min–max 14–24 %) [54]. In current practice, ET monotherapy provides a short PFS (<3 months), compelling the research of alternative therapeutic approaches [55].

### 3.1. Continuing CDK4/6i Therapy

Continuing CDK4/6i treatment beyond initial progression has been initially hypothesized as potentially effective in real-life retrospective studies, particularly in patients initially treated with palbociclib [56,57,58].

The randomized phase II MAINTAIN (NCT02632045) trial reported a significant 2.5 month improvement in PFS when continuing with a CDK4/6i (ribociclib) after progression in combination with a different ET (median 5.29 months vs. 2.76 months, HR 0.59; 95% CI 0.39–0.95, *p* = 0.006), compared to the switched ET + placebo arm; most patients had been previously treated with a different CDK4/6i (83% of the patients received palbociclib as the first CDK 4/6i). Data on safety and OS are not available yet. In an exploratory analysis, the benefit seems limited to *ESR1* wild-type (WT) in the fulvestrant subgroup. However, the *ESR1* mutant cohort was small, with a higher rate of *CCND1* and/or *FGFR1* gene amplifications; therefore, these data are hypothesis-generating [59]. Conversely, in the randomized phase II PACE trial (NCT03147287), no benefit was observed when continuing palbociclib (>90% continued palbociclib) with fulvestrant beyond progression on a prior CDK4/6i in terms of PFS (the median PFS was 4.6 months vs. 4.8 months, HR 1.11 (0.74–1.66)) or OS (the median OS was 24.6 months vs. 27.5 months, HR 1.02; 95% CI 0.67–1.56). In an exploratory analysis, patients who showed an ESR1 or PIK3CA mutation had a trend towards a PFS benefit when continuing palbociclib beyond progression [60].

Similarly, in the PALMIRA trial (NCT03809988), re-challenge with the CDK4/6i palbociclib plus ET did not improve PFS over ET alone in patients pretreated with palbociclib. The median PFS was 4.9 months in the treatment group versus 3.6 months in the control group (HR 0.84; 95% CI 0.66, 1.07; *p* = 0.149) [61] (Table 2).

It is likely that switching to a different CDK4/6i (as explored in the MAINTAIN trial) may be more successful than continuing with the same agent (as in the PACE trial), as differences in the mechanism of action and resistance between the various CDK4/6is can explain the different results between the three trials. In depth knowledge of tumor mutations and resistance mechanisms may allow the selection of patients who could benefit from continuing with CDK4/6is beyond progression.

### 3.2. Inhibitors of the PI3K/AKT/mTOR Signaling Pathway

Another strategy is to target other signaling pathways, such as the PI3K/AKT/mTOR signaling system. The PI3K/AKT/mTOR pathway is a critical signaling pathway involved in tumor growth, proliferation, and survival; its activation can promote resistance to ET.

*PIK3CA* somatic mutations are relatively early events in breast tumorigenesis and are present in approximately 30–50% of patients with HR^+^/HER2^−^ MBC [62]. PI3K inhibitors (particularly inhibitors of the PI3K-α isoform, which appear to be less toxic) have demonstrated potential in multiple clinical trials. In the phase III SOLAR-1 (NCT02437318) trial that investigated the addition of alpelisib (an inhibitor specific to the PI3Kα isoform) to fulvestrant, post-menopausal patients resistant to previous ET were enrolled, irrespective of their *PIK3CA* mutation status [39,63]. In patients with a mutation in the *PIK3CA* gene, the combination of alpelisib plus fulvestrant showed a significantly improved PFS in comparison with fulvestrant alone (11.0 vs. 5.7 months; HR 0.65; 95% CI 0.50–0.85; *p* = 0.00065), while there was no benefit for patients with wild-type forms. In the final OS analysis, although there was no statistically significant improvement in OS (HR 0.86; 95% CI, 0.64–1.15; *p* = 0.15), OS was prolonged to 7.9 months for those patients randomized in the alpelisib arm [63].

There was limited information available in the post-CDK4/6i setting, as only a small percentage (6%) of the population enrolled in the SOLAR-1 trial had been previously treated with a CDK4/6i. A large proportion of patients with disease progression after a CDK4/6i were included in the subsequent phase II BYLieve trial (NCT03056755); this study confirmed that the addition of alpelisib to ET is effective in patients with HR^+^/HER2^−^ MBC with a *PIK3CA* mutation who had previously been treated with a combination of CDK4/6is and ET [64].

Inavolisib is another PI3Kα-specific inhibitor that also promotes the degradation of mutant p110α, the catalytic subunit of *PIK3CA.* In the phase III INAVO120 trial (NCT04191499), the combination of inavolisib, palbociclib, and fulvestrant was associated with an improved PFS compared to palbociclib, fulvestrant, and placebo in patients with *PIK3CA*-mutated advanced tumors with disease progression within 12 months of completing adjuvant ET and no prior therapy for MBC (15.0 vs. 7.3 months, HR 0.43) [65].

The AKT pathway is located upstream from mTOR and downstream from PI3K and PTEN; activation of this sequence has been associated with endocrine resistance [66]. Early trials conducted on HR^+^/HER2^−^ MBC showed a clinically meaningful response to AKT inhibitors in combination with ET. The phase II FAKTION trial (NCT01992952), evaluating capivasertib (an AKT inhibitor) in combination with fulvestrant, provided statistically significant improvements in the PFS (10.3 vs. 4.8 months; HR 0.58; 95% CI 0.39–0.84; *p* = 0.004) and OS (HR 0.66; 95% CI 0.45–0.97; *p* = 0.035) of post-menopausal women with HR^+^/HER2^−^ MBC who experienced progression after or during aromatase inhibitor therapy (no prior CDK4/6i). An exploratory analysis showed that the benefit was limited to patients with mutated *PTEN, AKT1*, or *PIK3CA* genes [67].

The phase III CAPItello-291 (NCT04305496) clinical trial showed that capivasertib combined with fulvestrant had a significant effect on the median PFS that was doubled when compared to the placebo plus fulvestrant arm within the total research population (median PFS 7.2 vs. 3.6 months; HR: 0.60; 95% CI 0.51–0.71; *p* < 0.001); this included patients with and without mutations (unknown status included) in the AKT pathway (*PIK3CA*, *AKT1*, or *PTEN* genes) that had demonstrated progression after treatment with an aromatase inhibitor, with or without a CDK4/6i [68] (Table 3).

Other AKT inhibitors such as ipatasertib are being tested in ongoing trials (FINER) after failure under CDK4/6is and with progressive disease.

The mTOR signaling pathway is involved in malignant transformation and has roles in regulating apoptosis and cell proliferation [71,72]. Everolimus, a drug inhibiting the mTOR pathway, has been tested in combination with various endocrine agents. The first randomized trial to confirm the benefit of targeting this pathway in post-menopausal women with HR^+^/HER2^−^ MBC with progression on an aromatase inhibitor was BOLERO-2 (NCT00863655) [73]. The addition of everolimus to exemestane improved the median PFS (median PFS 10.6 vs. 4.1 months; HR 0.36; 95% CI 0.27–0.47), but showed no gain in OS [74]. However, no patients from the BOLERO-2 trial had previously been treated with CDK4/6is. Nonetheless, the administration of ET in combination with everolimus after disease progression during CDK4/6i treatment is substantiated by a large number of studies [75,76,77,78].

### 3.3. Fulvestrant and Novel Oral SERDs

SERDs are non-steroidal bifunctional molecules behaving both as competitive ER antagonists and inducing E3 ubiquitin ligase and the proteasome, causing the subsequent degradation of ER. With these dual mechanisms, they can escape tumor resistance, as shown by SERMs and AIs. Unlike SERMs, which can act as either agonists or antagonists, SERDs act exclusively as antagonists of ER. Fulvestrant is the first-class treatment for ER-positive MBC and is administered monthly as an intramuscular injection.

Fulvestrant has a much higher binding affinity for ER than tamoxifen (89% vs. 38%) [79]. By binding to ER, fulvestrant blocks the dimerization of ER and its nucleocytoplasmic transport [80]. Furthermore, the complex formed by ER and fulvestrant is unstable, which allows its degradation by the ubiquitin–proteasome system. Fulvestrant was initially approved as a 4-weekly 250 mg intramuscular injection, having shown not to be inferior to anastrozole in post-menopausal women with advanced BC who had progressed on first-line ET (tamoxifen was the first-line choice in the majority of cases at that time) [81,82].

In the phase III EFECT trial (NCT00065325), fulvestrant also achieved non-inferior results compared to exemestane for patients with progression after treatment with an aromatase inhibitor [83].

A high-dose regimen was subsequently established as the recommended schedule, and this consists of a loading dose of 500 mg administered three times at 14 day intervals and then every 28 days. This loading dose enabled steady-state plasma levels of fulvestrant to be reached within the first month of treatment [84]. The phase III double-blind placebo-controlled CONFIRM (NCT00099437) showed improved PFS and OS in patients receiving a dose of 500 mg compared to a dose of 250 mg of fulvestrant [85,86].

In the FALCON trial (NCT01602380), fulvestrant provided a significantly longer PFS than anastrozole in patients with advanced BC who had not received previous endocrine therapy (16.6 vs. 13.8 months) [16], and this finding allowed it to be approved in a first-line setting.

The efficacy of fulvestrant was reported in several randomized trials evaluating its combination with various biologic and targeted agents, such as CDK 4/6 inhibitors (PALOMA-3, MONALEESA-3, and MONARCH-2), alpelisib, and everolimus [15,16,18,39,87,88].

In phase III trials, fulvestrant has been often utilized as an ET partner with CDK4/6 inhibitors in both first- and second-line settings; however, its efficacy as a single agent after progression on CDK4/6 inhibitors explored as a control arm of several randomized trials remains limited, reaching a median PFS ranging between 1.9 and 4.7 months [59,89].

Molecular profiling might guide the selection of a SERD (fulvestrant or novel oral SERD) to obtain improved efficacy and overcome a recognized mechanism of acquired resistance to AIs, such as *ESR1* mutations. These mutations are uncommon in primary BC, but they are quite prevalent in metastatic disease, occurring in 25–40% of patients previously treated with AIs.

The data from the FERGI, SoFEA, EFECT, and PALOMA-3 trials were analyzed retrospectively to evaluate the impact of a mutation in *ESR1* on the tumor response to fulvestrant. The retrospective analysis from the phase III SoFEA trial highlighted a significantly longer PFS in those patients with *ESR1*-mutant MBC when treated with fulvestrant compared to exemestane (HR = 0.52; 95% CI 0.30–0.92; *p* = 0.02) [90]. According to these findings, patients with tumors presenting mutations in *ESR1* may benefit better from fulvestrant as an ET.

During first-line treatment with a CDK4/6i + ET, the early onset of *ESR1* mutations detected in circulating tumor DNA (ctDNA) could direct a switch strategy including modification of the endocrine backbone. Patients with ctDNA-detectable *ESR1* mutations who switched from an AI to fulvestrant (SERD) at the time of the early detection of the mutation and before radiological progression of the disease had a PFS of 11.9 months while on fulvestrant–CDK4/6i treatment when compared to 5.7 months on the AI-CDK4/6i treatment in the phase III PADA-1 (NCT03079011) study [91]. While these results are promising, it is yet unknown if the therapeutic adjustments made at the time the *ESR1* mutation develops, depending on radiographic progression, will affect survival.

However, a negative impact on the broad use and possible benefits of fulvestrant has been related to its bioavailability and its intramuscular formulation [92]. This led researchers to look for different orally accessible SERDs, a new family of more potent agents with a wider range of efficacy against *ESR1* mutations.

### 3.4. Novel Oral SERDs

The new orally accessible SERDs are non-steroidal molecules with an ER binding site and an ER degrading side chain with an amino acid or an acrylic acid base terminal. The development of *ESR1* mutations is an important ET escape mechanism, and it occurs in 25–40% of patients who have been treated previously with AIs or SERMs. BC bearing *ESR1* mutations usually shows resistance to additional AIs, but usually continues to be susceptible to SERDs.

The first potent agents with an acrylic acid side chain were all active metabolites of GW5638 (etacstil), namely, GW7604, GDC0810, AZD9496, G1T48, and LSZ102 [93,94,95,96]. However, the results of early-phase clinical trials with these agents showed poor efficacy and tolerability, and thus their development was interrupted. More active and tolerable SERDs, all of which have amino acid-based side chains, are currently undergoing investigation as a monotherapy post-CDK4/6i progression or in combination with CDK4/6is and other drugs, including immunotherapy or everolimus.

#### 3.4.1. Elacestrant

Elacestrant (RAD1901) is a basic amino side chain orally bioavailable SERM/SERD hybrid. It is a highly basic (pKa: 9.8) and lipophilic (cLogP: 6.8; LogD: 3.6) agent. In early clinical trials, it showed its safety, tolerability, good oral bioavailability, and its ability to penetrate the blood–brain barrier [97,98].

Moreover, it demonstrated a dose-dependent antitumor activity in the MCF-7 cell lines positive for ER and in xenograft models from patients with extensive treatment, including those resistant to fulvestrant and to CDK4/6is, and those with the Y537S and D538G *ESR1* mutations [99,100].

In a phase I study (NCT02338349), elacestrant at a dose of 400 mg once daily was safe and well-tolerated by post-menopausal women with heavily pre-treated (also with CDK4/6is) ER^+^/HER2^−^ MBC, including those with *ESR1* mutations (detected in 50% of patients); the objective response rate (ORR) was 19.4%, including 15% for patients previously treated with a SERD, 16.7% for patients who previously treated with CDK4/6is, and 33.3% for patients harboring a mutation in *ESR1* [101].

EMERALD (NCT03778931) is an international, randomized, multicenter, open-label phase III trial that enrolled a total of 477 post-menopausal women with ER^+^/HER2^−^ MBC previously treated with one or two previous lines of ET (including a CDK4/6i in all patients) and up to one prior line of chemotherapy. Patients were randomized to receive elacestrant or the standard of care (SOC), i.e., investigators’ choice of fulvestrant or an AI (exemestane, letrozole, or letrozole). Most patients in the SOC group were treated with fulvestrant (70%). *ESR1* mutations were detected in 47.8% of the patients, 43.4% had received two previous ET lines, and 20% had received chemotherapy for MBC. The co-primary endpoints were PFS in the overall population and PFS in the *ESR1* mutant subset.

A statistically significantly prolonged PFS was associated with elacestrant treatment in comparison with the SOC ET approach in the overall population (HR 0.70; 95% CI, 0.55 to 0.88; *p* = 0.002) and in patients harboring *ESR1* mutations (HR 0.55; 95% CI, 0.39–0.77; *p* = 0.0005) [102] (Table 4).

Although the absolute PFS benefit was small in this study (2.8 versus 1.9 months in the overall population and 3.8 versus 1.9 months in the *ESR1*-mutant population), the PFS curves significantly diverged at 6 months and 12 months after an initial dramatic drop in both treatment arms, perhaps as a result of rapid progression in patients who developed complete hormone resistance.

This finding implies that oral SERD therapy may still be beneficial for patients who retain some degree of hormone sensitivity after CDK4/6i treatment.

An update of the results demonstrated that the PFS benefit was greater (5.45 and 3.29 months; HR, 0.703) for the patients exposed for a longer time to CDK4/6is, especially in those who harbored mutations in *ESR1* (HR, 0.466; 95% CI, 0.270–0.791) [103,104]. A tendency to favor elacestrant in the overall population (HR 0.75; 95% CI: 0.54–1.04, *p* = 0.08) and in the population harboring *ESR1* mutations (HR 0.59; 95% CI: 0.36–0.96, *p* = 0.03), but not in the ESR1 non-mutant subgroup (HR 0.92; 95% CI: 0.59–1.42, *p* = 0.69), was shown in an interim OS analysis (149 deaths).

Based on these results, elacestrant has recently been approved by the FDA for use in post-menopausal women or adult men whose disease progressed after at least one line of ET and who had ER-positive, HER2-negative, *ESR1*-mutated advanced or metastatic BC. The most frequent side effect of elacestrant was nausea, reported by 35% of patients; grade 3 or 4 adverse events were modest. A proportion of 3.4% of the patients discontinued treatment with elacestrant when compared to 0.9% in the SOC arm.

#### 3.4.2. Camizestrant (AZD9833)

Camizestrant is another selective, non-steroidal, potent, pure antagonist of ERα oral SERD. It showed high antitumoral effects on xenografts (PDX) models derived from patients with ER^+^ BC, including those bearing a relevant mutation in *ESR1* [105].

The phase I multi-part SERENA-1 (NCT03616587) trial evaluated camizestrant both as a single agent or combined with a CDK4/6i (abemaciclib or palbociclib), an inhibitor of mTOR (everolimus), or an inhibitor of AKT (capivasertib) in ER^+^/HER2^−^ MBC patients. In a population with previous intensive treatment, camizestrant as a single agent showed an ORR of 10% with a median PFS of 5.4 months [106].

A combination of 75 mg of camizestrant and palbociclib demonstrated some clinical activity with an ORR of 5.9%, despite extensive pre-treatment, including chemotherapies, CDK4/6is, and fulvestrant [107].

In heavily pre-treated patients who received camizestrant combined with abemaciclib, the ORR was 26.3%, with a CBR at 24 weeks of 66.7%, while the median progression-free survival had not been reached [108].

The phase II SERENA-2 trial (NCT04214288) compared the safety and efficacy of camizestrant (in doses of 75 and 150 mg) with fulvestrant in post-menopausal women with advanced ER^+^/HER2^−^ BC who progressed after previous ET. Compared to EMERALD, those in this trial were less heavily pre-treated (prior to enrollment, no patient was allowed to have received more than one line of ET for MBC, and only 50% of patients had previously used CDK4/6 inhibitors; fulvestrant exposure was also absent). The treatment with camizestrant at both doses demonstrated a significant reduction in the rate of disease progression or death (for the 75 mg dose: PFS 7.2 vs. 3.7 months; 95% CI 0.41–0.81; HR 0.58; and for the 150 mg dose: PFS 7.7 vs. 3.7 months; 95% CI 0.48–0.92; HR 0.67) in the overall study population. There was a statistically significantly improved PFS in patients with *ESR1*-mutated disease, reducing the risk of progression or death by 67% at the 75 mg dose (PFS 6.3 vs. 2.2 months; 95% CI 0.18–0.58; HR 0.33) and by 45% at 150 mg dose (PFS 9.2 vs. 2.2 months; 95% CI 0.33–0.89; HR 0.55) The group of patients with wild-type *ESR1* receiving at least 12 months of ET+CDK4/6i therapy also showed statistically significant improved PFS over fulvestrant monotherapy [109] (Table 3).

Finally, at both the 75 mg and 150 mg doses, camizestrant reduced the amount of *ESR1* mutant ctDNA to undetectable or nearly undetectable levels.

Overall, camizestrant was well tolerated, and its safety profile was matched that of other studies. Photopsia was the most common treatment-emergent adverse event (TEAE); none of these occurrences, however, were grade 3 or higher. These findings from SERENA-2 suggest that according to EMERALD’s findings, patients with endocrine-sensitive disease and somatic *ESR1* mutations would benefit more from oral SERDs, perhaps due to a class effect.

Currently, SERENA-3 (NCT04588298) is a randomized, open-label, parallel-group, window-of-opportunity trial investigating the biological effects, safety, tolerability, and pharmacokinetics of camizestrant in different oral doses in the preoperative phase for post-menopausal women with primary BC.

A phase III randomized, multicenter, double-blind trial called SERENA-4 (NCT04711252) assesses the safety and effectiveness of camizestrant in combination with palbociclib in patients with ER^+^/HER2^−^ MBC without any systemic treatment for advanced disease.

The pivotal phase III SERENA-6 (NCT04964934) trial is a randomized, multicenter, double-blind, study assessing the switch to camizestrant versus continuing a non-steroidal AI in combination with a CDK4/6i (palbociclib or abemaciclib) in untreated patients with HR^+^/HER2^−^ MBC who have detectable mutations in *ESR1* on ctDNA during first-line treatment with an AI and CDK4/6 inhibitors. In the PADA-1 phase III trial, a similar approach—switching an AI for fulvestrant in patients who developed *ESR1* mutations—produced encouraging results.

The ongoing CAMBRIA-1 (NCT05774951) and CAMBRIA-2 (NCT05774951) clinical trials aim to evaluate the efficacy of camizestrant as adjuvant therapy in both switch and upfront treatment settings, respectively, and include patients also treated with adjuvant CDK 4/6 inhibitors.

#### 3.4.3. Amcenestrant

Amcenestrant promotes the shift of ERs into an inactive conformation while antagonizing the binding of estrogens to ERs.

Amcenestrant monotherapy has shown encouraging results in terms of the clinical benefit rate in the phase I AMEERA-1 trial (NCT03284957) [110]; the subsequent phase II AMEERA-3 trial (amcenestrant vs. ET as per physician’s choice) and phase III AMEERA-5 (amcenestrant/palbociclib versus letrozole/palbociclib) trial were halted at the interim analysis since there was no clinical benefit, although in AMEERA-3, a trend toward improved PFS was observed in patients with *ESR1*-mutated tumors [111]. These negative results, along with the recommendation of the Independent Data Monitoring Committee, led to the termination of the trials and of the global clinical development program, which included AMEERA-6 (NCT05128773) in the adjuvant setting [112] (Table 3).

#### 3.4.4. Giredenstrant

Another potent competitor of estradiol for ER binding is giredestrant, which induces its degradation.

In early phase trials, such as the open-label, multicenter, randomized, umbrella phase IA/IB GO39932 study (NCT03332797), giredestrant as a single agent (cohort A) and in combination with palbociclib (cohort B) had shown clinical activity with ORR and CBR (confirmed complete response, partial response, or stable disease ≥ 24 weeks) of 15% and 48%, respectively, and was well tolerated, with neutropenia as the most frequent side effect of the combination [113]. The efficacy and safety of giredestrant were compared with hormonal monotherapy in the randomized, open-label, multicenter phase II AcelERA (NCT04576455) trial, which enrolled patients with locally advanced or metastatic ER^+^/HER2^−^ disease who had previously received one or two lines of systemic therapy for metastatic disease (≤1 targeted therapy, ≤1 chemotherapy regimen, and prior fulvestrant allowed). After a median follow-up of 7.89 months, the study failed to meet the primary endpoint, namely, PFS (HR 0.81; 95%CI 0.60–1.10; *p* = 0.18) and has since been suspended. In patients with *ESR1*-mutant tumors harboring an *ESR1* mutations (39%), there was a non-significant trend for a benefit (median PFS 5.3 vs. 3.5 months; HR 0.60; CI: 0.35–1.03; *p* = 0.0610) [114] (Table 3).

In patients with HR^+^/HER2^−^ MBC, the phase III double-blind, placebo-controlled, randomized persevERA (NCT04546009) trial is now assessing the safety and effectiveness of giredestrant and palbociclib in comparison to letrozole and palbociclib in the first-line scenario.

In the early-stage (EBC) setting, giredestrant has also been studied, with encouraging results. Pre-operative giredestrant versus anastrozole was the treatment that post-menopausal women with untreated ER^+^ EBC and baseline Ki67 ≥ 5% were randomly assigned to receive in the phase II coopERA trial (NCT04436744); palbociclib was added to both arms after 14 days of only hormonal treatment (window-of-opportunity). The trial met its primary endpoint with a greater reduction in Ki67 from the baseline after 14 days on the single agent giredestrant in comparison with anastrozole: 80% (95% CI, 85–72%) vs. 67% (95% CI 56–75%; *p* = 0.0222) [115]. In the final analysis, patients treated with giredestrant + palbociclib sustained higher levels of Ki67 suppression (81%; 95% CI 86–75%) at the time of surgery compared to those receiving anastrozole + palbociclib (74%; 95% CI 80–67%) [116].

Lastly, giredestrant treatment vs. physician’s choice of ET will be evaluated for at least 5 years in the adjuvant setting in the randomized phase III lidERA trial (NCT04961996) of patients with medium- and high-risk ER-positive EBC.

#### 3.4.5. Imlunestrant (Ly3484356)

Imlunestrant is a selective ER degrader that causes sustained inhibition of ER-dependent gene transcription and cell growth. It has shown preclinical efficacy against BC models with wild-type and mutated *ESR1*, whether used alone or in conjunction with other targeted treatments.

The phase I/II EMBER-1 trial (NCT04188548) is evaluating the drug as a single agent and in combination with alpelisib, abemaciclib, everolimus, trastuzumab, or abemaciclib in post-menopausal and pre-menopausal patients with advanced ER^+^/HER2-negative BC or endometrial endometrioid cancer.

Preliminary results from heavily pre-treated patients with metastatic ER^+^/HER2^−^ BC showed a favorable safety profile and encouraging anti-tumor activity with an ORR of 12% and a median PFS of 4.3 months (range 3.6–7.1 months).

In patients progressing after a CDK4/6 inhibitor, using imlunestrant as a second-line treatment demonstrated a median PFS of 6.5 months (range 3.6–8.3 months), which was longer than expected using the currently standard mono-ET [117].

The EMBER-3 (NCT04975308) phase III trial is currently ongoing and will compare the effectiveness of imlunestrant alone vs. imlunestrant plus abemaciclib vs. the investigator’s choice of ET as a second-line treatment for patients who have not had chemotherapy or fulvestrant before (Table 5).

In the early-stage setting, several ongoing trials are studying the effects of imlunestrant in the neoadjuvant (EMBER-2, NCT0464748) and adjuvant (EMBER-4, NCT05514054) settings on patients at high risk of relapse.

## 4. Next-Generation Endocrine Agents

Primary and secondary resistance to classic ET drove the development of a new generation of anti-estrogen therapies, including new selective estrogen receptor modulators (SERMs), and novel therapies such as Complete Estrogen Receptor ANtagonists (CERANs), PROteolysis TArgeting Chimeras (PROTACs), selective estrogen receptor covalent antagonists (SERCAs), and selective human ER partial agonists (ShERPAs) [118]. Although some agents are in the early phases of clinical development, efficacy and tolerability data from phase III trials will further drive their introduction into clinical practice.

### 4.1. Complete Estrogen Receptor ANtagonists (CERANs)

CERANs bind to ER, causing its degradation and arresting its complete transcriptional activity. They can be used to avoid endocrine resistance in breast tumors thanks to their mechanism of inactivating both activation function 1 and 2 (AF1 and AF2), the two distinct transcriptional activation domains of ER. Normally, gene transcription and cell proliferation are induced by the double activation of AF1 and AF2: the first through several signaling pathways, such as mTOR, PI3K, and MAPK, with AF2 being activated by the estrogen ligand itself. SERDs and SERMs primarily deactivate AF2. CERANs have the unique capability to inactivate both AF1 and AF2 of ER by directly inhibiting the AF2 transcriptional activation domain and by recruiting nuclear receptor corepressors (N-CoR) to block AF1 [119]. In preclinical studies, palazestrant (OP-1250), an orally bioavailable CERAN, showed inhibition of both wild-type and mutant ER in breast cells. OP-1250 induced the shrinkage of both wild-type and mutant ER breast tumors in xenograft models [120].

Palazestrant (OP-1250)is under investigation in a phase I/II trial of heavily pretreated pre- and post-menopausal patients with HR^+^, HER2^−^ MBC; preliminary results demonstrated anti-tumor activity (ORR 9%, CBR 21%) and drug tolerability (the majority of TEAEs were grade 1–2, with nausea, fatigue, and constipation being the most prevalent) [121] (Table 5).

### 4.2. Novel SERMs and SERM/SERD Hybrids (SSH)

Tamoxifen is widely used in clinical practice for metastatic and adjuvant settings, being the first SERM approved in randomized phase III trials [122]. SERMs exhibit antagonist ER activity by inhibiting AF2 of ER, and agonist activity through AF1 across multiple signaling pathways, including mTOR, PI3K, and MAPK, depending on the type of cell, and through various co-activators and co-repressors. Other SERMs analogous to tamoxifen, such as raloxifene, toremifene, and arzoxifene, have been developed with different efficacy and safety results [123,124,125].

SERMs are classified based on their chemical structure and include SERMs (such as tamoxifen, toremifene, droloxifene, and idoxifene), benzothiophenes (raloxifene and arzoxifene), tetrahydronaphthalenes (lasofoxifene), and phenylindoles (SERM/SERD hybrids (SSHs): bazedoxifene and pipendoxifene) [126].

Toremifene showed similar efficacy to tamoxifen as an adjuvant treatment in post-menopausal operated ER+ BC patients [127,128,129].

Phase II trials showed interesting results for droloxifene and idoxifene in terms of the ORR in BC [130,131]. Lately, phase III trials showed no superiority of either drug as compared to tamoxifen, with similar toxicity profiles, leading to the discontinuation of the development of the molecules [132,133].

Raloxifene was initially studied for reducing the BC risk [134]. Unlike tamoxifen, raloxifene is not associated with an increased risk of endometrial cancer [70,124,135,136,137].

Despite showing modest efficacy in advanced BC [138], raloxifene’s efficacy in preventing BC [124,136,139,140,141,142,143,144] led to FDA approval (2007) to decrease the chance in postmenopausal women who have a high risk of developing BC or who have osteoporosis. [124]. As raloxifene has very low oral bioavailability [145,146,147,148], this has led to the development of arzoxifene, a benzothiophene SERM with better pharmacokinetics. Despite interesting in vitro efficacy [149], arzoxifene was inferior to tamoxifen in a phase III trial of untreated BC patients with respect to a TTP endpoint [150].

#### 4.2.1. Lasofoxifene

In preclinical models, lasofoxifene lowered the risk of developing HR^+^ BC by inhibiting growth of MCF7 Y537S and D538G primary tumors and metastasis to the lungs and liver [151].

In the phase II ELAINE-1 trial (NCT03781063) of patients with somatic ESR1-mutated HR^+^/HER2^−^ MBC (Y537S mutation in 40%) who progressed on prior AI and CDK 4/6 inhibitors, lasofoxifene produced a numerical but not statistically significant improvement in PFS compared with fulvestrant (6.04 vs. 4.04 months; HR 0.699, *p* = 0.138; the PFS at 12 months was 30.7% on lasofoxifene versus 14.1% on fulvestrant) [152].

The ELAINE-2 trial (NCT04432454) evaluated lasofoxifene combined with abemaciclib in heavily pretreated patients with *ESR1*-mutated MBC. The CBR was 65.5% (95% CI 47.3% to 80.1%). In patients with measurable lesions (18), the ORR was 55.6% (95% CI 33.7% to 75.4%). The median PFS was about 13 months. Lasofoxifene combined with abemaciclib was well tolerated, presenting primarily grade 1/2 treatment-emergent adverse events (TEAEs) [153].

A phase III study (ELAINE-3; NCT05696626) is ongoing and it is investigating the lasofoxifene + abemaciclib combination vs. fulvestrant + abemaciclib in patients with an *ESR1* mutation who progressed on palbociclib or ribociclib + AI and up to one line of chemotherapy in the metastatic setting [154] (Table 5).

#### 4.2.2. Bazedoxifene

Preclinical studies showed that bazedoxifene has antitumor activity in HR+ endocrine-resistant BC models [155,156], especially in the presence of Y537S *ESR1* mutations [157].

A phase Ib/II study showed an interesting CBR of 33.3% for bazedoxifene plus palbociclib in patients with HR+/HER2− advanced BC who progressed on prior ET, irrespective of ESR1 mutations [158].

### 4.3. Selective Estrogen Receptor Covalent Antagonists (SERCAs)

SERCAs inactivate ER by targeting a unique cysteine residue and inducing conformational changes without degrading it.

H3B-5942 is a SERCA that binds the cysteine residue at position 530 of both wild-type and mutant ERα, showing increased antagonist activity in cell lines. In xenograft models of BC, H3B-5942 demonstrated antitumor activity and superiority compared to fulvestrant. However, because H3B-5942 is dependent on covalent engagement, *ESR1* C530 mutations could serve as an escape mechanism in the therapeutic setting [159].

This issue led to the development of H3B-6545, a selective, orally available, small-molecule antagonist of ERα with an improved potency of the core scaffold, with the aim of enhancing the antagonist activity of the covalent link [160]. In preclinical studies, the novel drug was found to antagonize, as its predecessor, the wild-type and mutant ERα. Moreover, it was also found to be active in presence of the H524L and Y537S mutations, which seem to limit the activity of fulvestrant and raloxifene. In xenograft and tumor models, the HR3B-6545 was found to have greater antitumor activity compared to fulvestrant [160,161]. Thanks to these encouraging results, the small molecule was studied in a phase I/II trial (NCT03250676) enrolling 130 ER^+^, HER2^−^ MBC patients pretreated with CDK4/6 inhibitors (87%), fulvestrant (71%), or chemotherapy (54%). Of these, 58% had detectable *ESR1* mutations. In the phase I part, a dose of 450 mg once daily was established as the recommended phase II dose. A manageable safety profile was reported; the most common grade 2 AEs were anemia (20%), fatigue (16%), nausea (14%), diarrhea (11%), and increased AST levels (11%). Grade 4 AEs reported were considered related to disease progression. Sinus bradycardia of grade 1 and grade 2 were reported in 35 and 4% of cases. An ORR of 16.4%, a CBR of 39.7%, and a median PFS of 3.8 months were found in the preliminary analyses [162,163].

### 4.4. PROTACs

PROteolysis TArgeting Chimeras (PROTAC) are bifunctional molecules composed of two ligands joined by a linker that are able to recruit a specific target protein, such as ER, and bind to an E3 ubiquitin ligase.

A PROTAC induces ubiquitylation of the protein of interest and its subsequent degradation by the ubiquitin–proteasome system, after which the PROTAC is recycled to repeat the process.

The interaction between ER and ER3 ligase complex causes ubiquitylation of the target ER for proteasomal degradation [164].

PROTACs showed efficacy in HR+ BC, as they are able to degrade ER.

Vepdegestrant (ARV-471) is a first-in-class molecule that has demonstrated superior ER degradation and antitumor activity compared to fulvestrant in endocrine-sensitive and -resistant xenograft models [165].

In the phase I/II VERITAC clinical trial (NCT04072952), patients pre-treated with ETs and CDK 4/6is received vepdegestrant (ARV-471) dose escalation at a daily dose of 30 mg up to maximum dose of 700 mg. A maximum tolerated dose was not reached and no dose limiting toxicities (DLTs) were observed. Analyses of 12 paired biopsies from patients treated with 30 to 360 mg daily demonstrated up to 90% ER degradation in tumors expressing WT or mutant *ESR1*. This agent showed a manageable tolerability profile and a good clinical activity, producing a CBR of 41% in the overall population and 51% in patients with *ESR1*-mutated tumors [166,167].

The phase II dose expansion trial (Part B) of ARV-471 confirmed its CBR of 37.1% and 38.9% in the 200 mg and 500 mg doses, respectively, with a clinical benefit in ESR1-mutated subgroup (CBR of 47.4% and 54.5% in the 200 mg and 500 mg cohorts, respectively).

Part C of the study evaluated vepdegestrant in combination with palbociclib in pretreated HR^+^/HER2^−^ ABC patients and reported a good level of activity (CBR 63% in ITT population and 72.4% in *ESR1* mutant and mPFS = ~11 months in *ESR1* WT and *ESR1* mutant) [168].

Based on these results, the ongoing phase III study VERITACT-2 (NCT05654623) is comparing the efficacy and safety of 200 mg of vepdegestrant (ARV-471) vs. fulvestrant after progression on a first-line CDK4/6i plus ET, while another phase III study (VERITACT-3) in a first-line setting is comparing ARV-471 plus palbociclib and letrozole+ palbociblib.

ARV-471 is also being evaluated in an early setting in comparison with anastrozole in the randomized, non-comparative neoadjuvant phase II study TACTIVE-N [169].

AC682 is another orally available chimeric degrader of ER. Preclinical evidence demonstrated this agent’s effectiveness against tumor xenografts and in ER^+^ BC cells, including models with *ESR1* mutations. An ongoing phase I trial is currently examining the effects of AC682 on ER^+^ MBC (NCT05080842).

### 4.5. ShERPAs

ShERPAs are another new category of molecules showing promising results specifically in tamoxifen-resistant BC cells. Three ShERPAs were tested and validated in xenograft models of endocrine-independent and tamoxifen-resistant BC, and in contrast to agonists of ERα such as estradiol (E2), they did not cause significant uterine growth [170].

**Figure 2:**
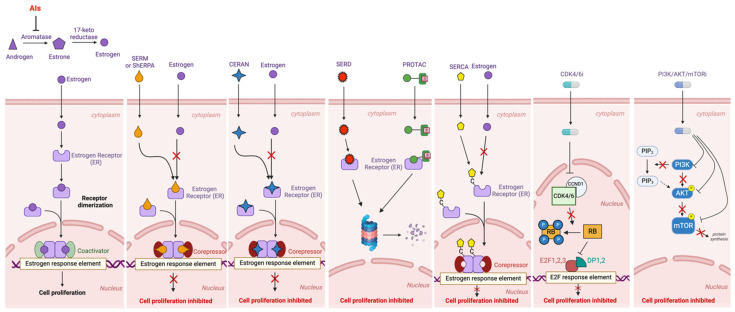
A scheme representing the different therapeutic strategies and the targets for ER^+^/HER2^−^ MBC refractory to first-line treatments. Created with BioRender.com.

We have outlined the therapeutic strategies to be potentially employed after progression on CDK4/6i therapy in Figure 2.

## 5. Other Potential Agents Targeting Components of the Cell Cycle

### 5.1. CDK7 Inhibitors

CDK7 is an enzyme comprising 346 amino acids; within the cell, together with cyclin H and MAT1, it constitutes the CDK-activating kinase (CAK) complex. It is able to facilitate the progression of the cell cycle through the phosphorylation of various CDKs and to promote gene transcription targeting the CTD of RNA polymerase II [171].

Considering the multiple roles of CDK7 in normal and cancer cells, namely to perpetuate their lineage, to give rise to differentiated cells, and to interact with their environment, it has been considered a target in drug-resistant human cancers and particularly TNBC [172].

Samuraciclib (ICEC0942) is a non-covalent, ATP-competitive, selective CDK7 inhibitor inducing cell cycle arrest and apoptosis in a preclinical study [173]. In HR^+^/HER2^−^ MBC patients who had previously progressed on an AI plus CDK4/6i, samuraciclib combined with fulvestrant showed a CBR of 36% [174]. Moreover, the CAK complex interacts with and activates p53, playing roles in the DNA repair process and, more generally, in tumor suppression [175].

Hence, CDK7 inhibitors could block aggressive luminal tumors that harbor *TP53* alterations.

### 5.2. SARMs

The androgen receptor (AR) is a steroid nuclear receptor frequently expressed in HR+HER2− BC (up to 95%) [176] and in 40–70% of ER-negative BC [177].

Selective androgen receptor modulators (SARMs) are small-molecule drugs that function as either AR agonists or antagonists. Variability in AR regulatory proteins in target tissues permits SARMs to elicit selective effects, preferentially stimulating bone and muscle growth, shrinking the prostate tissue, and inhibiting BC growth [178].

Co-culture signaling studies showed that SARM treatment inhibited the intra-tumor expression of genes and pathways promoting BC development and inhibiting its metastatic ability through the modulation of paracrine factors such as IL6 and MMP13 [177].

ARs may carry out divergent actions in ER^+^ vs. ER-negative BCs. AR agonism seems to exert a potent antitumor activity suppressing the growth of endocrine-sensitive and -resistant BCs [179].

Enzalutamide, a nonsteroidal antiandrogen, was combined with exemestane in a randomized phase II trial (NCT02007512) and failed to improve PFS compared to exemestane alone in ET-pretreated patients; in ET-naïve patients, high levels of AR mRNA were associated with a greater benefit of enzalutamide, particularly if low ESR1 mRNA levels were detected [180].

A recent study reported that the ratio of AR to ER in BC dictates the response to AR-targeted therapies, supporting the hypothesis of the clinical efficacy of enzalutamide in selected ER^+^ tumors with a low AR/ER ratio and AR agonists, such as RAD140, in tumors with a high AR/ER ratio [181].

In the open-label phase II G200802 trial (NCT02463032), enobosarm, a novel oral selective androgen receptor (AR)-activating agent, at a dose of 9 mg achieved a CBR of 32% in the 9 mg group and 29% in the 18 mg group. Additionally, the ORR was 48% in patients who had over 40% AR staining versus 0% with AR < 40% [182].

Currently, randomized phase III trials investigating enobosarm are ongoing (NCT04869943 and NCT05065411).

## 6. Final Considerations and Future Perspectives

In the management of HR^+^/HER2^−^ BC, ET targeting ER-driven disease still remains the mainstay treatment strategy either in the early or in advanced setting, including different options such as suppressing estrogen production (e.g., aromatase inhibitors, AIs, or luteinizing hormone-releasing hormone (LHRH) analogs) or directly blocking the ER pathway through SERMs—selective estrogen receptor modulators(e.g., tamoxifen)—or SERDs—selective estrogen receptor degraders (e.g., fulvestrant). The addition of CDK4/6is to ETs has significantly changed the therapeutic landscape, becoming the standard-of-care in the first- or second-line setting for advanced and high-risk early HR^+^/HER2^−^ BC. However, resistance to ET and CDK4/6is and tumor progression will inevitably occur. Unfortunately, there is no consensus on recommendations for therapeutic approaches in case of progression on the ET-CDK4/6i combination and, therefore, the optimal sequence continues to evolve in the rapidly changing treatment landscape. Some patients no longer have endocrine-sensitive disease after they have received CDK4/6 inhibitors, despite still having high ER or PR expression.

Thoroughly identifying resistance drivers could help define potential therapeutic strategies to overcome ET and/or CDK4/6i resistance such as the following:

(1) Continuing a CDK4/6i and switching to a different ET or re-challenging treatment with an alternative CDK4/6i;
(2) Targeting an altered ER pathway (fulvestrant, novel oral SERDs and other novel endocrine agents, including SERMs, SERCAs, CERANs, and PROTACs);
(3) Inhibiting alternative ER-independent signaling pathways (such as the PI3K/AKT/mTOR pathway, RAS/MAPK pathway, or tyrosine kinase receptors);
(4) Inhibiting pathways impacted by *BRCA1,2* germline mutations (PARP inhibitors).

The identification of key actionable genomic alterations in HR^+^/HER2^−^ MBC patients (somatic *ESR1* or PI3K/AKT/mTOR mutations or germline mutational drivers such as *BRCA1,2*) could drive the choice of tailored treatment (oral SERDs, alpelisib, capivasertib, or PARP inhibitors).

Mutations in *ESR1* contribute to ET resistance by mediating ligand-independent ER signaling and via constitutive ER activity; therefore, for these tumors, the ER pathway is still a promising therapeutic target.

BCs with *ESR1* mutations are typically resistant to further AIs, but they mainly remain susceptible to SERDs (i.e., fulvestrant) or novel oral SERDs (i.e., elacestrant).

However, polyclonal *ESR1* mutations may represent the cause of patients’ primary resistance to SERDs, indicating that subclones with concomitant genomic and epigenetic changes may also drive ER pathway-independent resistance [183].

In the phase III EMERALD trial, approximately 50% of the patients exhibited intrinsic ET resistance, showing rapid disease progression in both the experimental and control arms. Instead, a greater magnitude of benefit from elacestrant was observed among patients who had longer prior exposure to CDK4/6is (at least 12 months), especially *ESR1*-mutant patients. It is likely that patients who discontinue the use of a CDK4/6 inhibitor early in their disease course do not have a truly estrogen-driven disease.

Next-generation ETs, including SERMs other than tamoxifen, CERANs, SERCAs, and PROTACs targeting ER, are being currently developed to primarily overcome mechanisms of primary and secondary resistance to ET.

Noteworthy, *ESR1* mutations could have an important role in defining the treatment sequence in patients previously treated with CDK4/6 inhibitors.

Several second line trials, such as EMERALD, EMBER-3, AcelERA, and VERITAC-2, have reported interesting data on catching-up after *ESR1* mutation-driven disease progression. Noteworthy, the targeting of *ESR1* mutations as soon as they become detectable is being evaluated in the PADA-1 and SERENA-6 trials to personalize therapy selection in real-time. Most of these ER pathway inhibitors in combination with CDK4/6 inhibitors or other agents are being studied in first-line trials such as persevERA, SERENA-4, and VERITAC-3 to prevent *ESR1* mutation-driven resistance.

However, inconsistent results among different clinical trials with various agents from the same class (e.g., CDK4/6is and oral SERDs) suggest that not all drugs in the same class are similar or equally effective, although head-to-head comparisons have not been performed.

Other factors may influence the differing results between various studies, such as discrepancies in study populations and study designs (distinct rates of patients with de novo or recurrent metastatic disease; with a short or long disease-free interval, DFI; with primary hormonal resistance; previous treatment with multiple lines of hormone therapy; or with prior chemotherapy), missing survival data (patients lost to follow-up or consent was withdrawn, such as in the PALOMA-2 trial), limiting AEs, and the discontinuation rate (i.e., in adjuvant CDK4/6 inhibitor trials such as PALLAS and PENELOPE).

Indeed, the various individual agents have variable pharmacodynamics and, thus, different toxicities, affinities, mechanisms of action and resistance, and, ultimately, efficacies. The contribution of all factors mentioned above could potentially explain the differences in the outcomes of one study compared to other studies using different agents from the same class.

The ER-independent resistance mechanisms can arise by mutations or amplifications in membrane receptor tyrosine kinases (HER2, EGFR, and FGFR), alterations in the MAPK pathway (KRAS, BRAF, an dMAP2K1) and NF1, and the upregulation of the PI3K/AKT pathway, inducing less sensitivity to ET alone and requiring one or more combined targeted agents.

Alpelisib or capivasertib plus fulvestrant should be considered as a second-line therapy for patients presenting tumors with some alteration along the PIK3-AKT-mTOR pathway, considering the findings of the SOLAR-1 and CAPItello-291 trials.

For patients with germline *BRCA1,2* mutations, an oral poly (ADP-ribose) polymerase inhibitor (PARPi: olaparib or talazoparib) could be considered particularly as an alternative to chemotherapy, as defined in the OlympiAD (NCT02000622) and EMBRACA (NCT01945775) trials. They both demonstrated improvements in the ORR and PFS, but not in OS, for PARP inhibitors versus an investigator’s choice of chemotherapy regimen [184,185,186].

However, it should be underlined that there are currently no data on PARPi efficacy following CDK4/6i therapy because these trials were carried out before the advent of CDK4/6is. Furthermore, a number of studies indicate that patients with germline *BRCA1,2* mutations respond less well to first-line CDK4/6i treatment [187].

Finally, patients who rapidly progress on CDK4/6 inhibitors and ET (primary resistance), have no significant benefits from ET monotherapy or continuing CDK4/6is or inhibiting alternative ER-independent signal transduction pathways, while chemotherapy and eventually antibody–drug conjugates (ADCs) may be a better strategy [188].

In particular, in the phase III DESTINY-Breast04 trial (NCT03734029), trastuzumab deruxtecan (T-DXd), an antibody–drug conjugate (ADC) consisting of the monoclonal antibody trastuzumab with a topoisomerase I inhibitor payload, reported clinically relevant and statistically significant outcomes versus the physicians’ choice of chemotherapy in terms of the PFS and OS of patients who have progressed on prior ET (including CDK4/6is) and at least one line of chemotherapy with HR^+^/HER2^low^ disease (defined as tumors with an IHC score for HER2 between one and two, with no gene amplification) [189,190]. The impressive response rate and OS benefit recorded in the DESTINY-Breast04 trial suggest the rationale to use it earlier in patients with a severe disease load. T-DXd prior to chemotherapy is instead presently being investigated in the DESTINY-06 trial (NCT04494425).

Sacituzumab govitecan (SG) is another ADC that targets human trophoblast cell surface antigen 2 (TROP-2). In the multicenter phase III TROPiCs-02 study (NCT03901339), SG provided an OS benefit in patients with HR^+^/HER2^−^ MBC who progressed on a CDK4/6i, ET and at least two chemotherapy lines compared to the physicians’ choice of chemotherapy [191,192].

**Figure 3:**
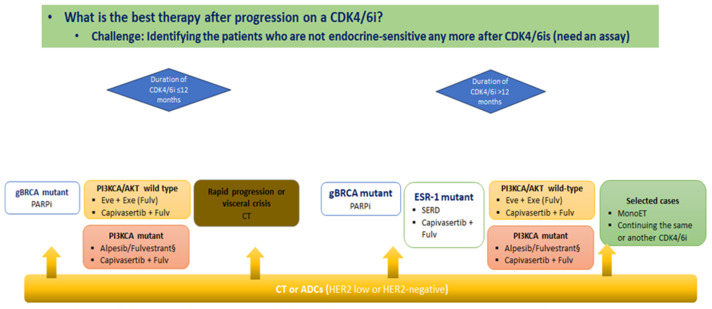
A potential algorithm for the treatment of HR^+^/HER2-negative MBC.

A current possible treatment algorithm for HR^+^/HER2-negative MBC disease after progression on a CDK4/6i is depicted in Figure 3.

New antibody–drug conjugates, novel therapies targeting the ER pathway, or other escape signaling pathways are currently being studied and will be included in the next therapeutic algorithms of HR^+^/HER2-negative disease. Therefore, further efforts are needed to understand the additional impacts of (i) tumor heterogeneity, (ii) the tumor microenvironment (stromal and immune cells), and (iii) intracellular crosstalk between these altered pathways to determine therapy resistance.

## Figures and Tables

**Table 1:** Phase III trials of CDK4/6 inhibitors involving endocrine-sensitive/resistant patients and their outcomes.

Study	PALOMA-2 [8,12]	MONALEESA-2 [5,9]	MONARCH-3 [7,11]	MONALEESA-7 [6,10]	PALOMA-3 [15]	MONALEESA-3 [16,17]	MONARCH-2 [18]
Setting	First-line	First-line	First-line	First- and second-line	Second-line	First- and second-line	Second-line
Endocrine therapy	Letrozole	Letrozole	Letrozole or anastrozole	Tamoxifen, letrozole, or anastrozole	Fulvestrant	Fulvestrant	Fulvestrant
CDK4/6 inhibitor vs. placebo	Palbociclib	Ribociclib	Abemaciclib	Ribociclib	Palbociclib	Ribociclib	Abemaciclib
No. of patients	666	668	493	672	521	669	726
ET status	Sensitive	Sensitive	Sensitive	Mixed	Resistant	Mixed	Resistant
PFS (months)	27.6 vs. 14.5	25.3 vs. 16	28.18 vs. 14.76	23.8 vs. 13.0	11.2 vs. 4.6	20.5 vs. 12.8	16.4 vs. 9.3
Hazard ratio (HR)	0.563 (0.46–0.69); *p* < 0.01	0.56 (0.45–0.70); *p* < 0.01	0.54 (0.44–0.69); *p* < 0.01	0.55 (0.44–0.69); *p* < 0.01	0.50 (0.40–0.62); *p* < 0.01	0.59 (0.48–0.73); *p* < 0.01	0.55 (0.44–0.68); *p* < 0.01)
OS (months)	53.9 vs. 51.2	63.9 vs. 51.4 (*p* = 0.004)	66.8 vs. 53.7	58.7 vs. 48.0	34.9 vs. 28.0	52.2 vs. 41.5 First line: 67.6 vs. 51.8	46.7 vs. 37.3
Hazard ratio (HR)	0.956 (0.777–1.177); *p* = 0.3378	0.76	0.804 0.804 (0.637–1.015) *p* = 0.0664	0.76 (0.61–0.96); *p* = 0.00973	0.81 (0.64–1.03); *p* = 0.09	0.754 (0.620–0.916); *p* = 0.004 First line: 0.67; (0.50–0.90)	0.75 (0.60–0.94); *p* = 0.01

**Table 2:** Therapeutic strategies after progression on CDK4/6i therapy: trials continuing CDK 4/6 inhibitors beyond progression.

Study	MAINTAIN [59]	PACE [60]	PALMIRA [61]
Phase	II	II	II
Study arms	Ribociclib and fulvestrant Placebo and fulvestrant	Fulvestrant Palbociclib and fulvestrant Palbociclib and fulvestrant and avelumab	Palbociclib and letrozole or fulvestrant Letrozole or fulvestrant
No. of patients	137	220	198
Primary endpoint ET status	PFS at 24 months	PFS at 24 months	PFS
mPFS (months)	5.29 vs. 2.76	4.6 vs. 4.8	4.9 vs. 3.6
Hazard ratio (HR)	0.59 95% CI (0.39–0.95); *p* = 0.006	1.11 95% CI (0.79–1.55); *p* = 0.62	0.84 95% CI (0.66–1.07); *p* = 0.149

**Table 3:** Therapeutic strategies after CDK4/6i resistance: main perspective trials targeting the PI3K/AKT pathway.

Study	INAVO 120 [65]	SOLAR-1 [63]	BYLieve [69]	CAPItello-291 [70]
Target Therapy	PI3K inhibitor	PI3K inhibitor	PI3K inhibitor	AKT inhibitor
Population		Progression on AI Stratification by prior treatment with CDK4/6is	Progression on no more than two previous anticancer therapies and no more than one previous chemotherapy regimen Confirmed *PIK3CA* mutation	Progression during or after treatment with an AI, with or without previous CDK4/6is
Prior CDK4/6i	PD within 12 months of completing adjuvant ET	Any	a. CDK4/6i + AI b. CDK4/6i + fulvestrant	Any
Subsequent TT	a. Inavolisib, palbociclib, and fulvestrant b. Palbociclib, fulvestrant, and placebo	a. Alpelisib + fulvestrant b. Placebo + fulvestrant	a. Fulvestrant + alpelisib b. Letrozole + alpelisib	a. Capivasertib + fulvestrant b. Placebo + fulvestrant
No. of patients	325	572	127	708
Efficacy	PFS (months) a. 15.0 b. 7.3 HR 0.43; 95% CI 0.32–0.59; *p* = 0.0001	PFS (months) a. 11.0 b 5.7. HR 0.65, 95% CI (0.50–0.85); *p* = 0.00065 mOS (months) a. 39.3 b. 31.4 HR 0.86; 95% CI 0.64–1.15; *p* = 0.15	PFS (months) a. 8.2 b. 5.6	PFS (months) a. 7.2 b. 3.6 HR 0.60; 95% CI 0.51–0.71; *p* < 0.001

Abbreviations: TT: targeted therapy; mOS: median overall survival; PFS: progression-free survival; PD: progressive disease; HR: hazard ratio; CI: confidence interval.

**Table 4:** Therapeutic strategies in the metastatic setting: oral SERD trials.

Study	EMERALD [102]	acelERA	AMEERA-3	EMBER (NCT04188548)	SERENA-2
Phase	III	II	II	III	II
N	477	303	282	830	288
Drugs	Elacestrant vs. ET (AI or Fulv)	Giredestrant vs. ET (AI or Fulv)	Amcenestrant vs. ET (AI or Fulv)	Imlunestrant vs. ET (AI or Fulv)	Camizestrant 75/150/300 mg vs. Fulvestrant
Number of prior lines	1–2	0–2	0–2	1	0–2
Previous chemotherapy	20%	allowed (≤1)	allowed (≤1)	allowed (≤1)	allowed (≤1)
% Previous fulvestrant	30%	allowed	allowed	not allowed	not allowed
% Previous CDK4/6i	100%	allowed	allowed (≤1)	allowed	allowed
mPFS (months)	PFS, ITT: 2.79 vs. 1.89; HR 0.7; PFS, *ESR1*mut: 3.78 vs. 1.87; HR 0.55	Did not meet the primary endpoint	Did not meet the primary endpoint	Not yet reported	PFS of the overall population: Fulvestrant 3.7 months. Camizestrant 75 mg, 7.2 months (HR 0.58; 95% CI 0.41–0.81; *p* = 0.0124) Camizestrant 150 mg, 7.7 mos (HR 0.67; 95% CI 0.48–0.92; *p* = 0.0161) PFS of the population with tumors harboring ESR1 mutations: Fulvestrant 2.2 months (95% CI, 1.9–3.6) Camizestrant 75 mg, 6.3 months (95% CI 3.4–12.9; HR 0.33; 95% CI 0.18–0.68) Camizestrant 150 mg, 9.2 months (95% CI 3.7–12.9; HR 0.55; 95% CI, 0.33–0.89)

**Table 5:** Novel anti-estrogen therapies: ongoing phase I–III trials.

Drug Class	Drug	Clinical Trial	Patient Population	N/State	Study Design	Endpoint	
SERD	Elacestrant	Phase Ib/II NCT05618613 (ELONA)	HR^+^/HER2^−^ MBC, with prior ET + CDK4/6i	Active, not recruiting	Elacestrant + onapristone		
	Elacestrant	Phase Ib/II NCT04791384	Post-menopausal, MBC with brain metastasis 1st/2nd line after prior CT	44	Elacestrant + abemaciclib	Overall intracranial response rate	
	Giredestrant	Phase III lidERA NCT04961996	Medium- and high-risk EBC	4100	Monotherapy vs. physician’s choice of ET	IDFS	
	Giredestrant	Phase Ib/II MORPHEUS NCT04802759	MBC 2nd/3rd line after progression on ET + CDK4/6i	415	Giredestrant + abemaciclib (alone or combined with atezolizumab), palbociclib, ribociclib, ipatasertib, inavolisib, everolimus, or samuraciclib	ORR	not yet reported
	Giredestrant (GDC-9545)	Phase III persevERA NCT04546009	HR^+^/HER2^−^ MBC 1st line	978	a. Giredestrant + palbociclib b. Letrozole + palbociclib	PFS	not yet reported
	Camizestrant	Phase III SERENA-4 NCT04711252	MBC 1^st^ line	1402	a. Camizestrant + palbociclib b. Anastrozole + palbociclib	PFS	
	Camizestrant	Phase III SERENA-6 NCT04964934	MBC, *ESR1* mutated with 1st line AI + CDK4/6i	1402	a. Camizestrant + CDK4/6i b. AI + CDK4/6i	PFS	
	Camizestrant	Phase II SERENA-3 NCT04588298	EBC without prior therapy	92	Single agent preoperatively	Change in ER expression	
	Imlunestrant	Phase I EMBER-2 NCT04647487	EBC without prior therapy	90	Single agent preoperatively	Change in ER expression	
	Imlunestrant (LY348356)	Phase Ia/Ib EMBER-1 NCT04188548	(Part A) up to 1L therapy MBC, no CDK4/6i (Part B) MBC with prior CDK4/6i	500	Imlunestrant (monotherapy) or combined with abemaciclib (+/− AI), everolimus, or alpelisib	DLT	
	Imlunestrant	Phase III EMBER-3 NCT04975308	Post-menopausal MBC with prior CDK4/6i	800	Imlunestrant (monotherapy) or combined with abemaciclib and physician’s choice of ET (fulvestrant/exemestane)	PFS	
	Rintodestrant	Phase I NCT03455270	MBC ≥ 2nd line	107	Rintodestrant + palbociclib	RPD2	
	D-0502	Phase I NCT03471663	MBC ≥ 2nd line	200	Monotherapy and combined with palbociclib	DLT	
	ZN-c5	Phase Ib 564TiP NCT04514159	MBC ≥ 2nd line, no prior CDK 4/6 inhibitor	14	ZN-c5 + abemaciclib	MTD	
	ZN-c5	Phase I/II 565TiP NCT03560531	MBC ≥ 2nd line	181	ZN-c5 + palbociclib	MTD	
	Borestrant	Phase I/II ENZENO NCT04669587	MBC any line	106	Monotherapy and combined with palbociclib	RPD2	
SERCA	H3B-6545	Phase II NCT04568902	MBC with at least 2 prior ET lines, or 1 prior ET and 1 prior CT, or 1 prior ET + CDK4/6i	94	H3B-6545	Efficacy of a single agent	
	H3B-6545	Phase I NCT04288089	Locally advanced/MBC ≥ 3rd line	36	H3B-6545 + palbociclib	MTD	
CERAN	OP-1250	Phase I/II NCT04505826	Locally advanced/MBC ≥ 2nd line 50% ESR1 mutated	94	OP-1250	MTD	
	OP-1250	Phase I NCT05266105	MBC	Recruiting	OP-1250 + palbociclib	Not reported	
	OP-1250	Phase Ib NCT05508906	MBC previously treated with ≤2 L of ET and 1 L of CT (prior CDK4/6i allowed)	Recruiting	a. OP-1250 + ribociclib b. OP-1250 + alpelisib	Not reported	
PROTAC	ARV-471	Phase I/II NCT04072952	MBC 2nd line (prior CDK4/6i allowed)	215	a. ARV-471 b. ARV-471 + palbociclib	DLT	
	ARV-471	Phase Ib NCT05501769	ABC with prior CDK4/6i	Recruiting	ARV-471 + everolimus	NA	
	ARV-471	Phase Ib TACTIVE-U NCT05573555	ABC treated with CDK4/6i, up to 2 L of prior therapies	Recruiting	ARV-471 + ribociclib	NA	
	ARV-471	Phase Ib/II TACTIVE-U NCT05548127	ABC ≥ 2nd line with prior CDK4/6i in any setting	Recruiting	ARV-471 + abemaciclib	NA	
	ARV-471	Phase III VERITAC-2 NCT05654623	ABC/MBC in progression on ET + CDK4/6i, with at least 6 months of ET prior to PD	Recruiting	a. ARV-471 b. Fulvestrant	NA	
SERM	Lasofoxifene	Phase II ELAINEII NCT04432454	MBC ≥ 2nd line (prior AI/CDK 4/6i required) with *ESR1* mutation			Safety (number and severity of AEs)	

Abbreviations: MBC—metastatic breast cancer; ABC—advanced breast cancer; EBC—early-stage breast cancer; AI—aromatase inhibitor; CT—chemotherapy; SERM—selective estrogen receptor modulator; SERD—selective estrogen receptor downregulators; PROTAC—PROteolysis TArgeting Chimeras; CERAN—Complete Estrogen Receptor ANtagonists; SERCA—selective estrogen receptor covalent antagonists; ORR—objective response rate; IDFS—invasive disease-free survival; PFS—progression-free survival; RPD2—recommended phase II dose; DLT—dose-limiting toxicity; MTD—maximum tolerated dose; ET—endocrine therapy; AEs—adverse events; NA—not available.

## Data Availability

No new data were created or analyzed in this study. Data sharing is not applicable to this article.
